# Unbiased CSF Proteomics in Patients With Idiopathic Normal Pressure Hydrocephalus to Identify Molecular Signatures and Candidate Biomarkers

**DOI:** 10.1212/WNL.0000000000213375

**Published:** 2025-02-14

**Authors:** Matthijs B. de Geus, Chao-Yi Wu, Hiroko Dodge, Shannon N. Leslie, Weiwei Wang, TuKiet T. Lam, Kristopher T. Kahle, Diane Chan, Pia Kivisäkk, Angus C. Nairn, Steven E. Arnold, Becky C. Carlyle

**Affiliations:** 1 Department of Neurology, Massachusetts General Hospital, Boston, MA;; 2 Leiden University Medical Center, the Netherlands;; 3 Harvard University, Boston, MA;; 4 Department of Psychiatry, Yale University, New Haven, CT;; 5 Janssen Pharmaceuticals, San Diego, CA;; 6 W.M. Keck Biotechnology Resource Laboratory, Yale School of Medicine, New Haven, CT;; 7 Department of Neurosurgery, Massachusetts General Hospital, Boston, MA;; 8 Broad Institute of Harvard and MIT, Boston, MA;; 9 Division of Genetics and Genomics, Boston Children's Hospital, MA;; 10 Department of Neurosurgery, Yale University, New Haven, CT;; 11 Picower Institute of Learning and Memory, Massachusetts Institute of Technology, Boston, MA;; 12 Department of Physiology Anatomy and Genetics, Oxford University, United Kingdom; and; 13 Kavli Institute for Nanoscience Discovery, Oxford, United Kingdom.

## Abstract

**Background and Objectives:**

Idiopathic normal pressure hydrocephalus (iNPH) is a reversible neurologic disorder that remains poorly understood. Accurate differential diagnosis of iNPH and Alzheimer disease (AD) is complicated by overlapping clinical manifestations. Beyond neuroimaging, there are currently no biomarkers available for iNPH leading to frequent misdiagnosis, and proteomic studies into iNPH have been limited by low sample sizes and inadequate analytical depth. In this study, we report the results of a large-scale proteomic analysis of CSF from patients with iNPH to elucidate pathogenesis and identify potential disease biomarkers.

**Methods:**

CSF samples were collected through lumbar puncture during diagnostic visits to the Mass General Brigham neurology clinic. Samples were analyzed using mass spectrometry. Differential expression of proteins was studied using linear regression models. Results were integrated with publicly available single-nucleus transcriptomic data to explore potential cellular origins. Biological process enrichment was analyzed using gene-set enrichment analyses. To identify potential diagnostic biomarkers, decision tree–based machine learning algorithms were applied.

**Results:**

Participants were classified as cognitively unimpaired (N = 53, mean age: 66.5 years, 58.5% female), AD (N = 124, mean age: 71.2 years, 46.0% female), or iNPH (N = 44, mean age: 74.6 years, 34.1% female) based on clinical diagnosis and AD biomarker status. Gene Ontology analyses indicated upregulation of the immune system and coagulation processes and downregulation of neuronal signaling processes in iNPH. Differential expression analysis showed a general downregulation of proteins in iNPH. Integration of differentially expressed proteins with transcriptomic data indicated that changes likely originated from neuronal, endothelial, and glial origins. Using machine learning algorithms, a panel of 12 markers with high diagnostic potential for iNPH were identified, which were not all detected using univariate linear regression models. These markers spanned the various molecular processes found to be affected in iNPH, such as LTBP2, neuronal pentraxin receptor (NPTXR), and coagulation factor 5.

**Discussion:**

Leveraging the etiologic insights from a typical neurologic clinical cohort, our results indicate that processes of immune response, coagulation, and neuronal signaling are affected in iNPH. We highlight specific markers of potential diagnostic interest. Together, our findings provide novel insights into the pathophysiology of iNPH and may facilitate improved diagnosis of this poorly understood disorder.

## Introduction

Idiopathic normal pressure hydrocephalus (iNPH) is a neurologic disorder characterized by abnormal CSF volume expansion in the ventricles and subarachnoid spaces,^1^ which is visible on radiologic imaging.^2^ Clinically, the iNPH syndrome is characterized by 3 main symptoms, gait impairment, urinary incontinence, and cognitive impairment, also called the Hakim-Adams triad of symptoms.^1^ A hallmark of iNPH is that diversion of excess CSF from the ventricular system, through placement of a ventriculoperitoneal shunt, can decrease or eliminate a patient's symptoms and neurologic deficits.^3^ Diagnosis is typically established by assessing the patient's main symptoms, characteristic imaging features, and their response to CSF diversion through a large-volume lumbar puncture (LP) or external lumbar drain.^4^

Differential iNPH diagnosis is complicated by overlapping symptomatology with other common conditions of aging, including arthritis; frailty; prostate and pelvic floor dysfunction; and common neurologic disorders, such as Alzheimer disease (AD), cerebrovascular disease, or Parkinson disease.^5,6^ Aside from neuroimaging, there are currently no biomarkers available for iNPH diagnosis, leading to frequent misdiagnosis in the form of both underdiagnosis and overdiagnosis.^7^ This may lead to excessive morbidity by not treating a treatable condition or unnecessary surgical intervention.

iNPH pathophysiology remains poorly understood, and studies into the underlying proteomic changes in iNPH are sparse and limited in sample size,^8^ or narrowly focused on inflammatory markers^9,10^ or micro-RNAs.^11^ Despite their limitations, these studies have suggested an upregulation of immune system–related markers. Other studies focusing on biomarkers that could help predict shunt response have identified markers related to brain injury, cell-cell adhesion, and blood-brain barrier (BBB) integrity and have indicated the involvement of specific glycoproteins, coagulation factors, and markers of immune response and astrocytic activation.^12-14^ Most work on fluid biomarkers has primarily focused on the role of typical AD amyloid and tau biomarkers, which can be useful in indicating comorbid AD pathology.^15,16^ More specific biomarkers encompassing distinctive molecular features of iNPH remain necessary for both improved diagnosis and understanding of pathophysiology.

We hypothesize that unbiased proteomics on clinically relevant samples will provide novel insights into iNPH pathophysiology and uncover potential biomarkers. In this study, we performed unbiased proteomics on a cohort of iNPH, AD, and neurologic control samples, systematically collected with consent for research use during patients' visits to a neurology clinic. We investigated between-group CSF proteome changes and identified relevant iNPH-associated biomarkers. We integrated these findings with publicly available tissue expression data to indicate their potential cellular origins. Then, we used ontology enrichment analyses to compare enriched biological processes between groups, highlighting distinct domains related to neuronal function, immune response, and vascular function that are perturbed in iNPH. Finally, we applied decision tree–based machine learning algorithms to illustrate pathophysiologic heterogeneity and highlight a set of potential iNPH diagnostic proteins. This study, leveraging patient samples from a real-world clinical setting, offers a unique insight into fundamental biological differences in iNPH compared with AD and cognitively unimpaired (CU) controls and provides candidate biomarkers for differential diagnosis.

## Methods

### Standard Protocol Approvals, Registrations, and Patient Consents

Samples were obtained according to standardized collection, processing, and research biobanking protocols of the Massachusetts General Institute for Neurodegenerative Disease. All participants provided written informed consent for research and biobanking (IRB: 2015P000221), and all activities were performed according to ethical standards of the Declaration of Helsinki.

### CSF Collection and Processing

All CSF samples were obtained through LP or external lumbar drains in the Neurology clinic at the Massachusetts General Hospital, according to standardized protocols based on established dementia clinical cohorts.^17^ For patients with iNPH, positive LP response was defined by pregait/postgait assessments through a 3-meter timed-up-and-go test. All samples were collected in the morning between 8 am and 12 pm without fasting. Samples were visually checked for blood contamination and spun down at 2000g for 10 minutes. Within 60 minutes after LP, samples were aliquoted and put on dry ice before long-term storage at −80 °C. All samples were stored in the same freezer with constant monitoring and temperature recording for 20–66 months. AD biomarkers, amyloid-β_42/40_ ratio, p-tau^181^, and total tau were assessed using Euroimmun ELISAs (Euroimmun, Lubeck, Germany). AD status was corroborated with an Aβ_42/40_ ratio below the in-house threshold of 0.0818 (sensitivity 91.6%, specificity 91.3%).

### Mass Spectrometry

Mass spectrometry (MS) methods have been described previously.^18^ To reduce technical variability in MS acquisition related to sample complexity, total protein concentration was normalized to 7.5 µg in 50 µL of ammonium bicarbonate. Proteins were digested in 2 steps using 1:20 LysC:sample protein followed by 1:20 trypsin. Data-independent acquisition (DIA) liquid chromatography mass spectrometry was performed in 20 batches of 24 samples, with all samples injected in duplicate plus a batch-to-batch technical control sample at the start and end of each batch. MS data acquisition was performed in DIA mode with isolation windows of 25 m/z, with the full scan ranging from 400 to 1,000 m/z. Protein identification and quantification were performed using DIA-NN (version 1.8.1),^19^ using an in silico generated library on the Uniprot Homo Sapiens database (UP000005640, 80581 sequences, 14.10.2022). This generated a data set of 576 unique protein groups expressed in at least 80% of all samples with coefficients of variation between technical replicates below 25%. To attenuate for batch effects, data were normalized with the *ComBat()*algorithm from the sva package (version 3.46.0)^20^ and visualized using principal component analysis (eFigure 1).

### Statistical Analysis

Statistical analyses were performed in R studio (R version 4.2.2). Protein abundances from DIA-NN were log-transformed, z-scored, and scaled between 0 and 1 for analyses. After quality control, batch-to-batch technical control sample data were excluded from further analysis. A small but significant age difference between groups was observed (*p* < 0.05, Table); therefore, age was included as a covariate in subsequent analyses. A linear model was fit using the *lm()* function, including age and sex as covariates: *lm(relative protein abundance ∼ diagnostic group + age + sex)*. The *tidy()* function (broom package version 1.0.4) was used to extract *p* values, and adjusted *p* values were calculated using the Benjamini-Hochberg method.

**Table:** Demographics and ATN Biomarkers by Cognitive Status

	N	Sex	Age		Aβ_42/40_ ratio	pTau181	Total tau
		Female, N (%)	Mean (SD)	Range	Mean (SD)	Mean (SD)	Mean (SD)
CU	53	31 (58.5)	66.5 (6.98)	58–89	0.11 (0.014)	26.1 (6.58)	194.7 (50.6)
AD	124	57 (46.0)	71.2 (7.45)	58–85	0.05 (0.012)^a,b^	114.9 (43.5)^a,b^	541.5 (222.9)^a,b^
iNPH	44	15 (34.1)	74.6 (6.87)	60–94	0.11 (0.019)	23.3 (15.7)	171.0 (72.4)

Abbreviations: AD = Alzheimer disease; ATN = Amyloid, Tau, Neurodegeneration; CU = cognitively unimpaired; iNPH = idiopathic normal pressure hydrocephalus.

Between-group differences in continuous variables were assessed using *t* tests. There was a significant age difference between groups (*p* < 0.05). Participants with AD had significantly increased levels of pTau181 and total tau and a lower Aβ_42/40_ ratio compared with CU and iNPH groups. No significant differences were observed for the ATN biomarkers between the CU and iNPH groups.

a Significantly different compared with iNPH (*p* < 0.05).

b Significantly different compared with CU (*p* < 0.05).

### Single-Nucleus RNA-Sequencing Integration

To assess the potential cellular origin of the changes observed, publicly available human single-nucleus sequencing data^21^ were integrated with the linear model results. A normalized mean gene enrichment score was calculated:

$$\log10\left(\frac{raw\;gene\;expression\;in\;a\;cell\;type}{mean\;gene\;expression\;across\;all\;cell\;types}\right)$$

Normalized scores were plotted using the *Heatmap()* function from the ComplexHeatmap package. Cell-type data available in this data set included vascular leptomeningeal cells, pericytes (Peri), endothelial cells (Endo), oligodendrocytes (Oligo), astrocytes (Astro), microglia (Micro), oligodendrocyte precursor cells, excitatory neurons (Exc), and inhibitory neurons (Inh).

### Gene-Set Enrichment Analysis

Gene-set enrichment analysis (GSEA) was used to gain understanding of the biological differences between groups. Gene-set enrichment analysis uses a ranked list of features and calculates an enrichment score for defined Gene Ontology (GO) terms from multiple databases. For this study, we looked at “biological process” GO terms, which group together functionally related proteins according to their role in a specific biological module or program.^22^ The coefficients from the linear regression model comparing protein abundances between groups were used as the ranked input. The GSEAs were performed for each group comparison on STRING-db^23^ using the functional enrichment analysis tool and searched against the homo sapiens database. Both area under the fold change curve and Kolmogorov-Smirnov methods of calculating enrichment scores were used. A false discovery rate cutoff of 1% was used, and significantly enriched processes were compiled. Overlap between GO processes was illustrated by calculating a mean protein abundance of all proteins annotated to each term for each participant. Biological pathway analysis on the Kyoto Encyclopedia of Genes & Genomes (KEGG) database was performed with the *enrich_kegg()* function from the bioconductor package.

### Decision Tree Models

To identify the proteins that best discriminate groups, we used decision tree analyses. First, we applied a full-sample decision tree to the whole data set (3 groups) to visualize the underlying decision-making process. The full-sample decision tree was generated using the rpart package in combination with the tidymodels package. rpart implements the Classification and Regression Trees algorithm, constructing binary decision trees by recursively splitting the data into subsets based on the feature that results in the greatest improvement in the classification accuracy. The resulting decision tree was visualized using the *rpart.plot()* function (rpart.plot package).

Next, we ran 5 iterations of 10-fold cross-validated random forest (RF) models to identify proteins with high diagnostic potential. We designed four two-way models between the diagnostic groups. RF models were fitted using the ranger algorithm (caret package^24^). The model was trained to predict the diagnostic group based on all proteins in the data set, including age and sex. The *train()* and *traincontrol()* functions from the caret package^24^ were used for model training. Hyperparameters were tuned using a grid search approach with the number of variables randomly sampled at each split ranging from 1 to the total number of predictors in the data set, incremented by 20. The Gini importance score was used as the node-splitting rule. The Gini importance indicates how much a feature improves the decision-making process in a RF. If a feature is important, it will be used more often in the splits that more effectively separate different classes, leading to a higher Gini importance score. The minimum terminal node size was set to 1. Receiver operator characteristic (ROC) curves of the top identified proteins from the RF analysis were generated using the *roc()* function (pROC package). A composite ROC score for the top 6 proteins from each two-way contrast was created using a generalized linear model.

### Data Availability

Normalized protein abundance scores are given in eTable 1. R scripts are available at github.com/ACTRU/iNPH-CSF-Proteomics. Underlying raw data have been deposited to ProteomeXchange through MassIVE (PXD043216).

## Results

### Study Cohort

**Figure 1:**
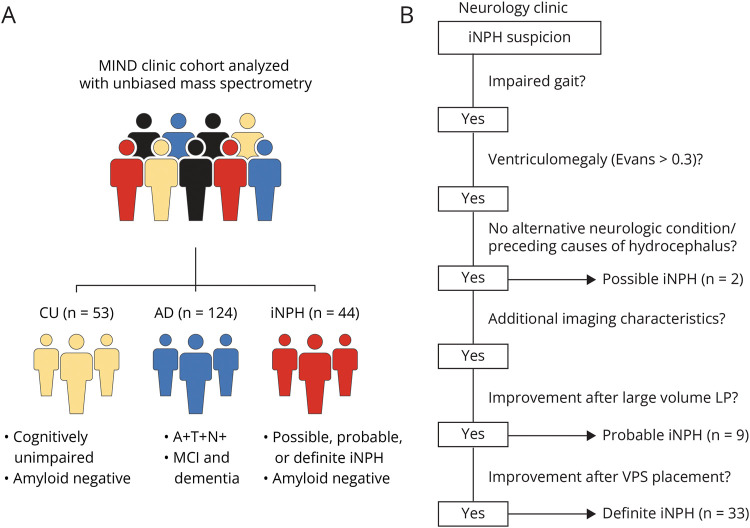
Cohort Overview and Clinical iNPH Diagnosis Overview (A) Study samples were selected from the Massachusetts General Institute for Neurodegenerative Disease (MIND) cohort and analyzed with mass spectrometry. Participants were subdivided into cognitively unimpaired (CU) controls, Alzheimer disease (AD) cohort, and idiopathic normal pressure hydrocephalus (iNPH) cohort. (B) iNPH diagnosis was established through a systematic assessment in the MassGeneral NPH Program according to guidelines of the Japanese Society of Normal Pressure Hydrocephalus. Possible iNPH is diagnosed when the following features are present: (1) clinical symptoms of gait impairment with or without other classic triad symptoms (bladder symptoms, cognitive impairment); (2) ventriculomegaly on neuroimaging as defined by an Evans index equal to or greater than 0.3; (3) clinical symptoms that cannot be better explained by alternative neurologic or other conditions, for example, parkinsonism, cerebrovascular, orthopaedic, and myelopathic; (4) no other obvious preceding causes of hydrocephalus (e.g., aqueductal stenosis, meningitis, and subarachnoid hemorrhage). Probable iNPH is diagnosed with (1) requirements of possible iNPH and (2) either (a) presence of additional neuroimaging features including vertex crowding, acute callosal angle, and/or disproportionately enlarged subarachnoid hydrocephalus (DESH), or (b) objective improvement in gait after large-volume lumbar puncture or external lumbar drain. Definite iNPH is diagnosed when there are both: (1) requirements met for possible iNPH and (2) definite and objective improvement in signs/symptoms after ventriculoperitoneal shunt surgery. Clinical response to shunt was routinely determined 2 to 3 weeks after surgery with assessment of gait as the primary outcome, as well as bladder and cognitive symptoms. A total of 44 patients with iNPH, comprising possible, probable, and definite iNPH, were included in the study. In addition, all patients with iNPH were negative for AD biomarkers.

CSF samples were collected during patients' diagnostic evaluation visits to the Neurology clinic at Massachusetts General Hospital. All participants had a clinical indication for an LP. The following comparison groups were selected based on demographics, clinical diagnosis, and CSF AD biomarker results (Table, Figure 1A):

1. Cognitively unimpaired (CU = 53) was defined by having unimpaired cognition and negative AD CSF biomarker results to remove participants with “asymptomatic AD.” This group included participants who were being evaluated for immune disease and patients with other nondementing neurodegenerative diseases, vascular disease, demyelinating disease, headache, psychiatric disease, idiopathic intracranial hypertension, neoplasm, or no other specified neurologic disorders (e.g., history of positive serum VDRL).
2. AD (N = 124) included participants with either mild cognitive impairment or dementia who tested positive on all 3 CSF AD biomarkers.
3. iNPH (N = 44) was ascertained based on documented clinical and neuroradiologic profiles with the application of systematic criteria for diagnosis of iNPH, adapted from the Guidelines of the Japanese Society of Normal Pressure Hydrocephalus^25^ (Figure 1B, eTable 2). The detailed diagnostic criteria for possible, probable, and definite iNPH are presented in the footnote of Figure 1. To exclude comorbid AD pathology, possibly confounding our comparative analyses, we further required all iNPH cases to have an amyloid-β_42/40_ above the in-house established cutoff for AD.

The AD group showed elevated levels of both total tau and pTau^181^ and a lowered amyloid-β_42/40_ ratio compared with both CU and iNPH (Table). No difference in AD biomarkers was observed for iNPH compared with CU.

### Differentially Expressed Proteins

**Figure 2:**
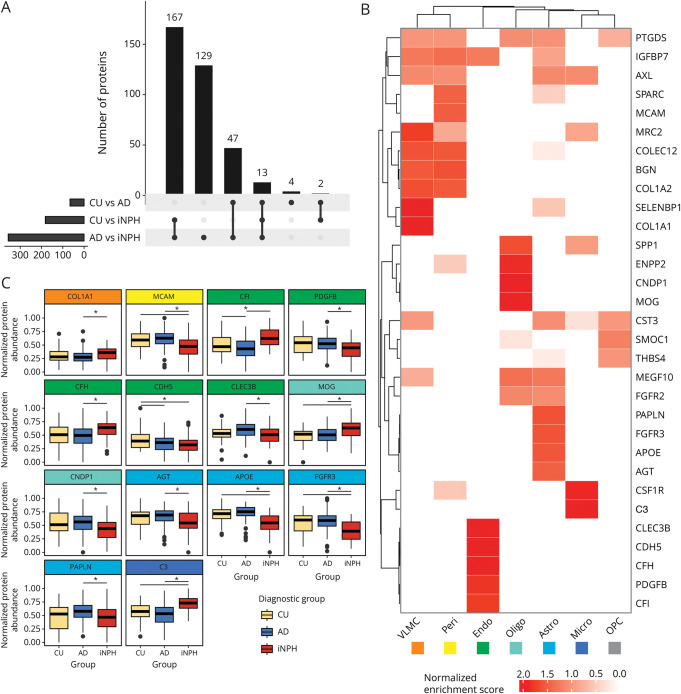
Differential Expression Analysis and Integration With Transcriptomic Data (A) Upset plot indicating the number of differentially expressed (DE) proteins, from the linear regression model output, between groups and how many proteins were significant in 1 (single dot) or more (joined dots) contrasts. A large number of proteins were found to be DE in both CU vs iNPH and AD vs iNPH, indicating a broader dysregulation of the proteome in iNPH compared with AD and CU. (B) Integration with publicly available transcriptomic data indicates DE proteins from a likely specific cellular origin. DE proteins that showed a normalized gene expression score >1 (i.e., an enrichment of at least ten-fold in 1 cell type vs all others) are visualized in a heatmap across non-neuronal cell types. Dendrograms indicate hierarchical grouping between proteins and cell types. (C) Expression profiles of DE proteins that were found to be highly enriched in a single cell type. Boxplots indicate the median and interquartile range, and black points indicate outliers. For example, collagen, type 1, α 1 (COL1A1), increased in iNPH compared with AD, was highly enriched in vascular leptomeningeal cells. Significance bars indicate Benjamini-Hochberg adjusted *p* < 0.05 from linear regression model results. AD = Alzheimer disease; CU = cognitively unimpaired; iNPH = idiopathic normal pressure hydrocephalus.

Proteomic differences between diagnostic groups were explored using simple linear regression models (eTable 3). Of the 576 identified proteins meeting quality control criteria, 356 were differentially expressed (DE) between iNPH and AD, 182 between iNPH and CU, and 66 between AD and CU (Benjamini-Hochberg adjusted *p* value <0.05; Figure 2A). Overall, more proteins were downregulated in iNPH, with 248 downregulated proteins of 356 DE proteins compared with AD and 161 of 182 DE proteins compared with CU (eFigure 2). There were 167 DE proteins that overlapped when comparing either CU or AD with iNPH, 47 DE proteins that overlapped when comparing either CU or iNPH with AD, and 13 DE proteins that were different between all 3 contrasts (Figure 2A). Furthermore, 129 DE proteins were unique to the AD and iNPH comparison. Together, these numbers suggest a distinct proteomic profile in iNPH.

### Integration of RNA-Sequencing Data Reveals Potential Cellular Origins of DE Proteins

We investigated potential cellular origins of the differential protein expression data. DE proteins from the linear regression models were integrated with publicly available single-nucleus RNA-sequencing data.^21^ A normalized enrichment score was calculated for each protein in each cell type compared with all cell types, with a score of 1 indicating a ten-fold enrichment of a gene in 1 cell type compared with all others. A full analysis of DE proteins across all cell types is presented in eFigure 3. Most of the DE proteins in iNPH were expressed in excitatory and inhibitory neurons, but a number were strongly enriched in non-neuronal cell types (Figure 2B). Figure 2C shows the direction of change of selected proteins observed in CSF across the different diagnostic groups. Together, contextualizing proteomic data with single-nucleus transcriptomic data indicates a broad variety of potential cellular origins of the proteomic changes observed in iNPH.

### GSEA Unveils Distinct Biological Processes in iNPH

**Figure 3:**
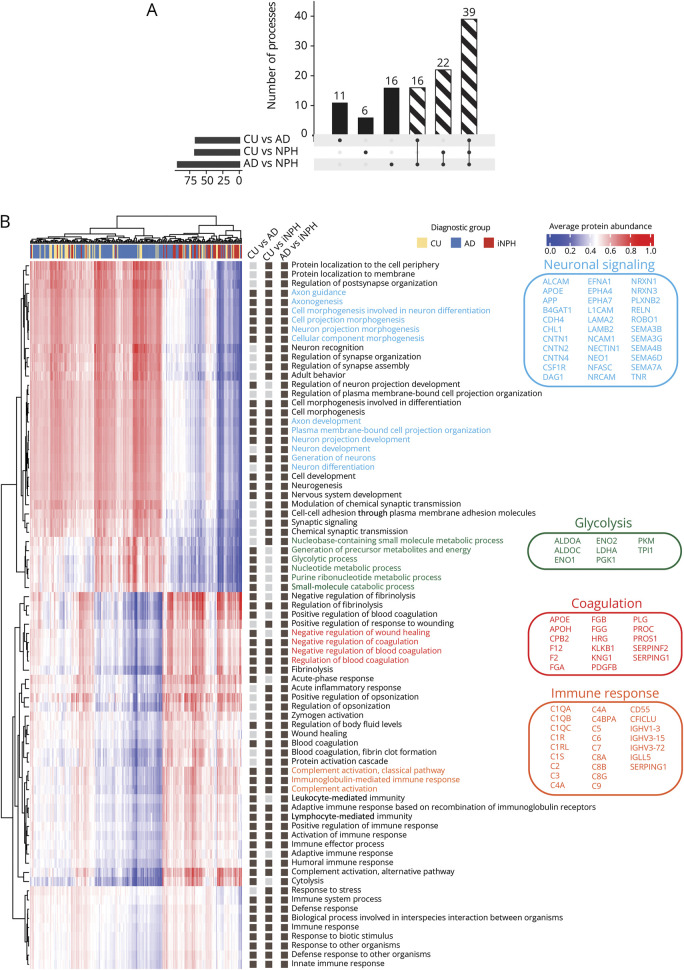
Pathway Analysis Indicates Altered Immune Response and Synaptic Signaling in iNPH (A) Upset plot showing the number of “*biological processes*” Gene Ontology terms enriched in each contrast after GSEA analysis. Dashed bars indicate the number of processes that are significant in multiple contrasts; for example, 39 processes are significant in all 3 contrasts. (B) Heatmap of the average protein abundance per GO term for each participant. Dendrograms indicate hierarchical clustering of participants along the x-axis and GO terms along the y-axis. Participant clustering indicates a clear upregulation of coagulation and immune system–related processes in participants with iNPH. In addition, processes related to neuronal signaling and synaptic integrity were downregulated in iNPH. Processes related to cellular metabolism were specifically upregulated in AD compared with both CU and iNPH. The dark gray boxes to the right of the heatmap indicate significant enrichment of the process in the corresponding contrast. Common proteins driving enrichment of related GO terms are listed on the right-hand side and colored to match GO term labels on the heatmap. GO = Gene Ontology; GSEA = gene-set enrichment analysis; iNPH = idiopathic normal pressure hydrocephalus.

To investigate underlying molecular differences in iNPH compared with AD and CU, we applied GSEA using the linear model coefficients of the proteins. Only the GO terms corresponding to the “*biological process*” ontology were used (eTable 4). When comparing iNPH with AD, there were 93 terms significantly enriched (Figure 3A). Furthermore, 67 terms were significantly enriched when comparing iNPH with CU and 66 terms between CU and AD. Of these, 22 were enriched when comparing either CU or AD with iNPH, indicating that these biological processes are uniquely affected in iNPH. 16 terms were overlapping when comparing either CU or iNPH with AD and 39 terms overlapping between all 3 contrasts.

Using the significantly enriched terms between the different groups in two-way comparisons, we illustrated the patterns of change for these processes between all groups. The average protein abundance of all proteins annotated to each term was calculated, creating an average term abundance for each participant, visualized in the heatmap in Figure 3B. Results for each two-way contrast are shown in eFigures 4–6. Processes that were downregulated in participants with iNPH compared with both AD and CU groups were mostly driven by neuronal transmembrane proteins such as semaphorins, neurexins, or contactins, annotated to GO terms of neuronal development, neuronal signaling, or cell-cell interactions. Consistently upregulated processes in iNPH compared with both AD and CU were mostly driven by complement factors and immunoglobulins annotated to GO terms of immune system processes, such as complement activation or immunoglobulin-mediated response. Processes related to vascular biology, such as regulation of fibrinolysis and blood coagulation, also seemed upregulated in iNPH compared with AD and CU and were driven by fibrinogen proteins and coagulation factors. When comparing AD with both CU and iNPH, the enriched processes predominantly relate to various aspects of cellular metabolism, such as small-molecule metabolism and glycolytic metabolism. Proteins annotated to metabolic processes were upregulated in AD compared with CU and iNPH, suggesting an upregulation of cellular metabolism in AD, which has been described before.^18,26^ Many of the significant processes were driven by clusters of the same proteins (Figure 3B). Synaptic processes downregulated in iNPH seemed to be driven by neuronal adhesion molecules such as *neural cell-adhesion molecule 1*, *neurexin 1*, *neurexin 3* (NRXN3), and axon guidance proteins from the *semaphorin* family. Metabolic processes upregulated in AD were mostly driven by glycolytic enzymes such as *aldolase A* (ALDOA), *enolase 1* (ENO1), *phosphoglycerate kinase 1*, *pyruvate kinase* (PKM), and *lactate dehydrogenase A* (LDHA). Vascular coagulation processes upregulated in iNPH were driven by coagulation factors and fibrinogen markers. Immune system–related changes were driven by members of the complement activation cascade. iNPH-enriched processes were validated using the KEGG database (eFigure 7).

### Decision Tree Models Illustrate Pathologic Heterogeneity Between and Within Diagnoses

**Figure 4:**
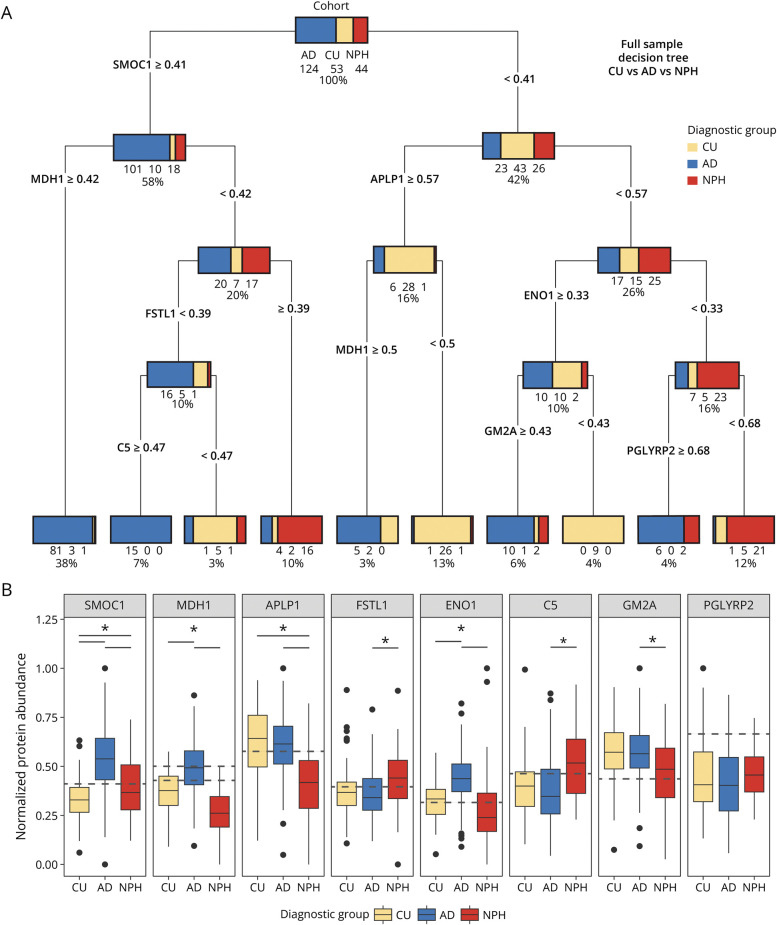
Full-Sample Decision Tree Highlights Heterogeneity Across Diagnoses (A) Full-sample decision tree model used for classifying samples into iNPH, CU, and AD. The root node at the top represents the entire data set, with branches splitting according to the best discriminatory proteins. Terminal nodes display the final classification with the percentage of samples in each group. Most AD cases were identified based on high SPARC-related modular calcium-binding protein 1 (SMOC1) levels. However, even with high levels of SMOC1, the model still identified some CU and iNPH cases based on levels of malate dehydrogenase 1 (MDH1), follistatin like 1 (FSTL1), and complement protein 5 (C5) proteins. Most CU and iNPH cases showed lower levels of SMOC1. Instead, distinctions were made based on levels of amyloid-β precursor-like protein 1 (APLP1), MDH1, enolase 1 (ENO1), peptidoglycan recognition protein 2 (PGLYRP2), and ganglioside GM2 activator protein (GM2A). (B) Boxplots showing the abundances of the proteins used in the classifying splits in the decision tree model. Dashed lines indicate the decision thresholds determined by the full-sample decision tree model. These thresholds represent the specific protein abundance values at which the model differentiated between cohort groups during classification. The placement of these dashed lines within the distribution of protein abundances visualizes the alignment of the decision-making criteria with the data. Black points show outlier measurements. The significance bars indicate significance levels from linear regression results, *Benjamini-Hochberg adjusted *p* < 0.05. AD = Alzheimer disease; CU = cognitively unimpaired; iNPH = idiopathic normal pressure hydrocephalus.

To investigate markers of potential diagnostic interest in the data set, we used a full-sample decision tree model (Figure 4A). The model consisted of 5 layers with 9 decision nodes and 10 leaf nodes, with 5 leaf nodes belonging to AD, 3 to CU, and 2 to iNPH, with an accuracy of 87.8%. The different ways this decision tree model comes to the classification of the 3 diagnostic groups indicates the heterogeneity in the disease population and potential differential underlying pathology.

A benefit of these decision tree–based models is their ability to identify nonlinear and multivariate effects in data sets. In Figure 4B, the relative abundances for the proteins the decision tree used as deciding features for its classification process are shown, with dotted lines indicating the abundances used for the model splits. Linear regression analyses between groups for each protein indicated significant group differences for some proteins. *SPARC-related modular calcium-binding protein 1* (SMOC1) was found to be significantly different between all 3 groups (Benjamini-Hochberg adjusted *p* value <0.05). *Malate dehydrogenase 1* (MDH1) and ENO1 were found to be significantly upregulated in AD compared with both CU and iNPH. *Amyloid-*β *precursor like protein 1* was significantly lower in iNPH compared with CU and AD. *Follistatin like 1* and *complement protein 5* were found to be higher in iNPH while *ganglioside GM2 activator* was found to be lower in iNPH compared with the other groups. The effect of *peptidoglycan recognition protein 2* (PGLYRP2), as indicated by the full-sample decision tree, is not detected by linear regression models, indicating the benefits of these decision tree models in finding potential diagnostic markers between groups.

### Random Forest (RF) Models Highlight Potential Diagnostic Markers of iNPH

**Figure 5:**
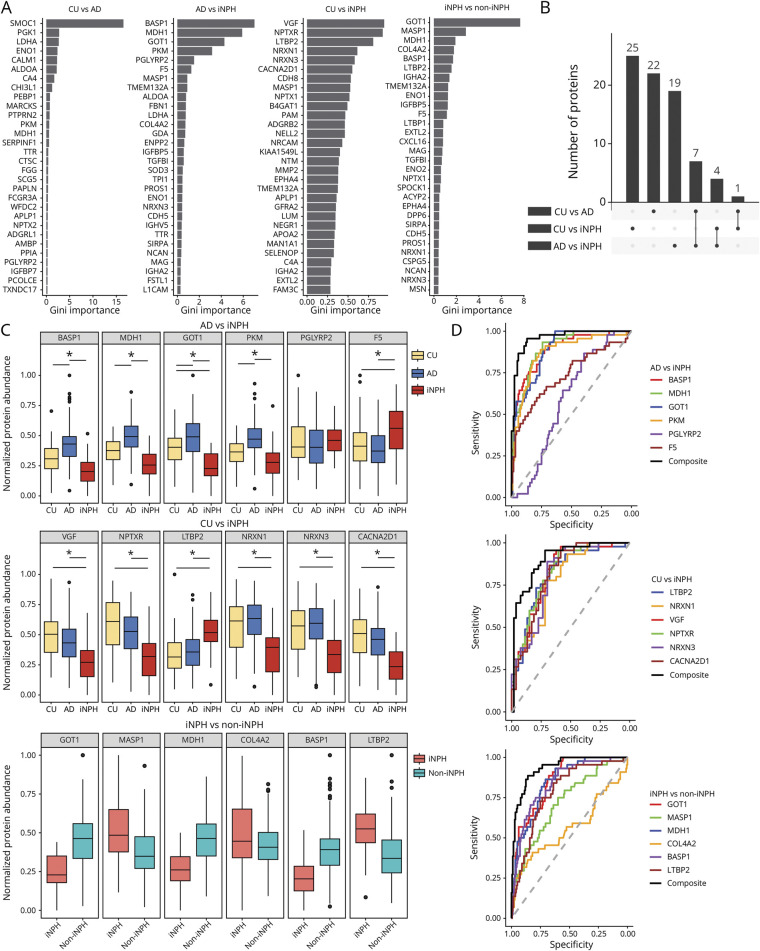
Random Forest Models Indicate Potential Diagnostic Panel of Proteins for iNPH (A) Gini importance of the top 30 proteins from each two-way random forest model, indicating top classification potential within each model. For example, the CU vs AD contrast was driven mostly by SMOC1. (B) Upset plot showing whether the top 30 proteins in each model were important in only 1 model (single dot) or multiple models (joined dots). 7 proteins were important in both AD contrasting models and 3 proteins overlapped in iNPH contrasting models. A heatmap showing the abundances of these proteins is shown in eFigure 8. (C) Protein abundances of the top 6 important proteins in the iNPH contrasting models. The top 6 proteins of the iNPH vs non-iNPH model showed high overlap with the other models except for the inclusion of mannose-binding lectin-associated serine protease 1 (MASP1) and collagen type IV α 2 chain (COL4A2). Significance bars indicate linear regression model results; *Benjamini-Hochberg adjusted *p* < 0.05. (D) ROC curves showing diagnostic potential of top 6 proteins from iNPH contrasting models. GLM-based composite scores of proteins combined outperformed each individual protein indicating combined diagnostic relevance of these proteins. AD = Alzheimer disease; CU = cognitively unimpaired; iNPH = idiopathic normal pressure hydrocephalus.

To define potential diagnostic proteins of iNPH with higher predictive certainty, we applied cross-validated RF models to our data set. Three separate two-way RF models were run for comparisons between all groups. Each model showed a high predictive accuracy between groups of 77.5% (CU vs iNPH), 83.5% (AD vs iNPH), and 84.1% (CU vs AD). In addition, a model was fit comparing iNPH with all non-iNPH participants together, which yielded an accuracy of 85.6%. However, it is important to note that the larger group imbalance in this comparison (44 iNPH vs 177 non-iNPH) could affect model performance. The top 30 proteins with the highest classification capability from each model are shown in Figure 5A. Classifications in the AD vs CU and AD vs iNPH models were primarily driven by the top 6 proteins as indicated by a high Gini importance. Gini importance rapidly decreased across the other top proteins. Conversely, the CU vs iNPH classification seemed to be driven by smaller contributions of more proteins. These top proteins represent a wide variety of molecular and biological functions spanning glycolytic enzymes, synaptic proteins, coagulation factors, and immune system–related proteins. Figure 5B shows the overlap between the top proteins from each model indicating that there are several proteins that share importance between models. Seven proteins were overlapping between CU vs AD and iNPH vs AD, which could indicate markers more indicative of AD LDHA, ENO1, ALDOA, PKM, MDH1, PGLYRP2, and *transthyretin*. Four proteins, *mannan-binding lectin serine protease 1*, *transmembrane protein 132A* (TMEM132A), *immunoglobulin heavy constant* α *2*, and NRXN3, were overlapping between CU vs iNPH and AD vs iNPH, potentially indicating markers more indicative of iNPH (eFigure 8). Figure 5C shows the directions of change for the top 6 important proteins from the 2 iNPH contrasting models as well as the combined non-iNPH vs iNPH contrast. Linear regression results indicate that GOT1 was significantly different between all 3 groups, suggesting divergent underlying pathology between AD and iNPH. *Brain acid soluble protein 1* (BASP1), MDH1, and PKM all showed AD-specific upregulation while synaptic proteins NPTXR, NRXN3, and VGF were all significantly downregulated in iNPH compared with both AD and CU. F5 and LTBP2 were found to be significantly upregulated in iNPH compared with CU and AD.

To investigate the diagnostic predictive accuracy of the top 6 proteins from the iNPH contrasting models, ROC curves were used (Figure 5D, eFigure 9). Individually, the top proteins of the AD vs iNPH contrast had a varying predictive potential with areas under the curve (AUCs) spanning from 0.84 for BASP1 and MDH1 to 0.59 for PGLYRP2. When using a generalized linear model (GLM)-based composite of these top proteins together, a higher AUC of 0.96 was achieved. When leaving PGLYRP2 out of this composite, the AUC dropped to 0.95. Similarly, when comparing CU and iNPH, a GLM-based composite of the top 6 proteins outperformed the individual proteins with an AUC of 0.90. Further analysis also showed that markers from the same biological processes were correlated with each other (eFigure 9). Together, these results indicate the potential value of decision tree–based models to indicate a panel of proteins with diagnostic capability for iNPH.

## Discussion

iNPH is a reversible neurologic disorder where the underlying pathophysiology remains remarkably underexplored. In this study, we used unbiased CSF proteomics in a real-world clinical cohort to elucidate mechanistic differences in patients with iNPH compared with patients with AD and CU individuals and highlight a panel of potentially relevant molecular biomarkers to aid in diagnosis. iNPH is characterized by the enlargement of CSF spaces in the brain. In this study, most of the DE proteins were decreased in abundance in CSF in iNPH. A potential reason for these large decreases may be a dilution effect of all CSF contents. Although we show that substantially more proteins were decreased in iNPH, the total protein content of all samples was normalized before analysis by MS. Previous studies have shown no differences in total protein concentration in CSF from patients with iNPH.^27,28^ It is possible in our study that the general downregulation of many proteins observed in iNPH does not change total protein content because of significant upregulation of immune proteins.

**Figure 6:**
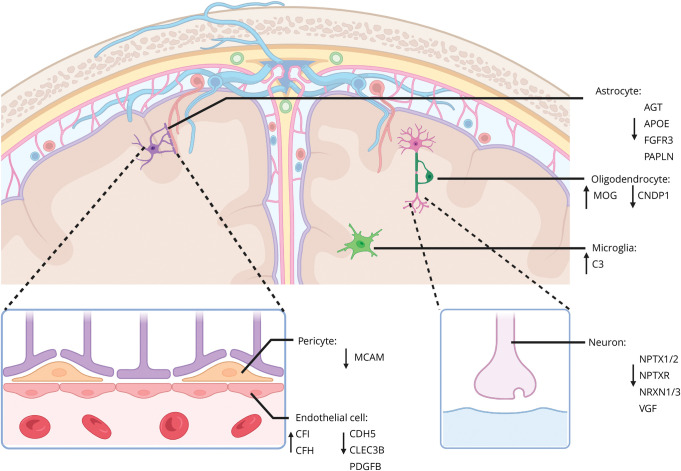
Schematic Overview of Observed Changes in iNPH and Their Potential Cellular Origin Proteins that were observed to have a significant increase or decrease in CSF in iNPH compared with either AD or CU. A decrease in synaptic markers was observed and a decrease in endothelial and pericyte markers, potentially indicating impaired synaptic signaling and impaired blood-brain barrier (BBB) integrity, respectively. This impaired BBB integrity could manifest as a leaky BBB that could lead to the extravasation of coagulation and immune system markers observed to be upregulated in iNPH. An increase in complement system markers in microglia and endothelial cells is indicative of a heightened immune response in iNPH. Created in BioRender. De Geus, M. (2025) BioRender.com/c79r194. AD = Alzheimer disease; CU = cognitively unimpaired; iNPH = idiopathic normal pressure hydrocephalus.

Some of the most strongly downregulated proteins identified in iNPH comprised processes related to neuronal signaling or synaptic integrity (Figure 6). Some markers driving these changes included key synaptic adhesion and transmission markers such as neurexin proteins, neuropentraxin proteins, and VGF. Sequence variation in the neurexin family of proteins has been associated with a wide variety of neurologic and psychiatric disorders,^29^ changes in *neuropentraxin 2* have been associated with the cognitive dysfunction in AD,^30,31^ and abnormal VGF has been a robust finding and even proposed as a therapeutic target for neurodegenerative diseases.^32^ Together, these indications of faulty synaptic integrity could form the basis of the cognitive impairment in iNPH, but the underlying mechanism and correlation with reversal of symptoms after shunt placement warrant further investigation.

We observed a downregulation of the endothelial cell adhesion marker CDH5 in iNPH. Endothelial cells of the brain's vasculature together with pericytes and astrocytic endfeet constitute the BBB. The permeability of the BBB is integral to its function of controlling the exchange of molecules and cells from the blood to the brain parenchyma.^33,34^ This permeability is controlled by adherens junctions, of which CDH5 forms an essential component.^35^ We can speculate that the decrease in CDH5, observed here in iNPH, may reflect impaired BBB integrity. Leakage of the BBB has been shown to lead to the extravasation of fibrinogen,^33^ and we show an upregulation of various fibrinogen subunits, fibrinogen alpha chain, fibrinogen beta chain, and fibrinogen gamma chain, but also other coagulation markers F2, F5, and F12. Increased permeability of the BBB may also be tied to our observed increase of immune system–related markers. We found a broad upregulation of immune system markers from the complement cascade and immunoglobulins. Changes in the immune system have been reported before in general hydrocephalus, and specific immune system–related markers have been studied as biomarkers of iNPH.^10,36,37^ A leaky BBB has been shown to lead to extravasation of immune markers in iNPH.^33^ Whether changes in immune system markers play a role in the pathophysiology of iNPH, either as cause or consequence of associated BBB leakage, should be investigated further.

The molecular changes in iNPH, observed here, should also be considered in the context of the glymphatic system. Despite its recent discovery, it has been identified as a crucial paravascular system for removing toxic waste from interstitial fluid to the CSF.^38,39^ Its functioning is essential to maintaining biochemical homeostasis in the brain, and a dysfunctional glymphatic system has been associated with iNPH and AD.^40,41^ The aquaporin-4 (AQP4) water channels, located in the astrocytic endfoot, are integral to the flow of CSF, and AQP4 has been proposed as a therapeutic target.^42,43^ AQP4 has been shown to be reduced in iNPH, leading to a diminished exchange of the interstitial fluid and CSF. The observed decreases in a vast number of proteins in CSF could arise from an impaired efflux of these proteins from the interstitial fluid into the CSF. The mechanism of interaction between the markers identified to be decreased in CSF and AQP4 demands additional exploration.

Using machine learning models, we identified a panel of proteins that offer diagnostic biomarker potential for iNPH. These proteins encompass all the various biological modalities that we showed to be affected in this disease. With this multivariate nonlinear approach, we found some proteins, for example, PGLYRP2, that would be missed through conventional linear approaches. We showed that accurate differentiation of iNPH from AD was mostly dependent on the abundance of glycolytic enzymes, a known characteristic of AD.^18^ Differential diagnosis of iNPH from controls seemed more dependent on synaptic markers. Our models also validated the diagnostic importance of the established AD marker, SMOC1, in AD compared with controls, highlighting the reliability of our approach. This marker has been associated with amyloid plaque pathology,^44^ indicating its relevance in AD but not for iNPH. Combining these proteins into a diagnostic panel, for example, through targeted MS, may provide significant clinical relevance for physicians who could rely on more objective measures in the diagnostic analysis of iNPH.

The cohort used in this study consisted of clinical patients who were retrospectively selected based on specified criteria rather than a prospectively defined case-control study. This approach enhances the real-life applicability of our findings by reflecting the typical heterogeneity seen in clinical practice, but the lack of a meticulously matched healthy control group may introduce confounding factors that could influence the results. To account for these differences, we included age and sex as covariates in our statistical models. In addition, the neurologic control group used here was made up of patients who had an indication for an LP but were otherwise cognitively unimpaired and were AD biomarker negative. The choice of a cohort relevant to a diagnostic setting means that there are individuals with inflammatory and metabolic changes in the control group. This may mask some changes in iNPH that would be evident with a control group consisting of individuals with no neurologic indication, such as an orthopaedic surgery group. Clinical improvement after shunt surgery was assessed using routine evaluations by the treating clinician rather than objective scales, potentially introducing subjective bias.

In conclusion, this study leverages data from a true-to-life clinical cohort to provide a comprehensive overview of the proteomic changes observed in iNPH compared with AD and neurologic controls. We validate key biological domains specifically affected in iNPH and highlight key markers of these processes. Finally, a novel panel of potential diagnostic markers is proposed for further evaluation of iNPH.

## Glossary

AD: Alzheimer disease
ALDOA: aldolase A
AQP4: aquaporin-4 water channel
AUC: area under the curve
BASP1: brain acid soluble protein 1
BBB: blood-brain barrier
CU: cognitively unimpaired
DE: differentially expressed
DIA: data-independent acquisition
ENO1: enolase 1
GLM: generalized linear model
GO: Gene Ontology
GSEA: gene-set enrichment analysis
iNPH: idiopathic normal pressure hydrocephalus
KEGG: Kyoto Encyclopedia of Genes & Genomes
LDHA: lactate dehydrogenase A
MASP1: mannan-binding lectin serine protease 1
MDH1: malate dehydrogenase 1
MS: mass spectrometry
NPTXR: neuronal pentraxin receptor
NRXN3: neurexin 3
PGLYRP2: peptidoglycan recognition protein 2
PKM: pyruvate kinase
RF: random forest
ROC: receiver operator characteristic
SMOC1: SPARC-related modular calcium-binding protein 1
